# Human Immune System Mice With Autologous Tumor for Modeling Cancer Immunotherapies

**DOI:** 10.3389/fimmu.2020.591669

**Published:** 2020-10-08

**Authors:** Liguang Sun, Chun-Hui Jin, Shulian Tan, Wentao Liu, Yong-Guang Yang

**Affiliations:** ^1^ Key Laboratory of Organ Regeneration & Transplantation of the Ministry of Education, The First Hospital of Jilin University, Changchun, China; ^2^ National-local Joint Engineering Laboratory of Animal Models for Human Diseases, Jilin University, Changchun, China; ^3^ Department of Pathology, The First Hospital of Jilin University, Changchun, China; ^4^ International Center of Future Science, Jilin University, Changchun, China

**Keywords:** humanized mouse, cancer, immunotherapy, CAR T cell, human immune system mouse, allogeneic hematopoietic cell transplantation

## Abstract

Mouse models are the most commonly used *in vivo* system for biomedical research, in which immune-related diseases and therapies can be investigated in syngeneic and immunologically intact hosts. However, because there are significant differences between rodent and human, most findings from conventional mouse models cannot be applied to humans. The humanized mouse with a functional human immune system, also referred to as human immune system (HIS) mouse, is the only model available to date for *in vivo* studies in real-time of human immune function under physiological and pathological conditions. HIS mice with human tumor xenografts are considered an emerging and promising *in vivo* model for modeling human cancer immunotherapy. In this review, we briefly discuss the protocols to construct HIS mice and elaborate their pros and cons. Particular attention is given to HIS mouse models with human tumor that is autologous or genetically identical to the human immune system, which are discussed with examples of their usefulness in modeling human cancer immunotherapies.

## Introduction

Human immune system (HIS) mice have been highly instrumental for *in vivo* studies of human immune function and immune disorders. The HIS mouse was the first model that made it possible to study human immune responses in real time *in vivo* under physiologic or pathogenic conditions, such as HIV pathogenesis (1), human xeno-immune responses (2), complex interplay between hypercholesterolemia and human adaptive immunity (3), and intrathymic selections of human T cells (4). HIS mice, conjunct with tissue chimeras (i.e., with organ repopulation by human parenchymal cells), were found highly valuable in elucidating immunopathology of human-tropic viral infections, such as hepatitis B and hepatitis C viruses (5, 6) and respiratory viruses (7). HIS mice were also increasingly used in the studies of human cancer immunology and immunotherapy. However, most of the models used in these studies were either immunocompromised or involving allogeneic and/or xenogeneic immune responses, making the host immune environment different from that of patients. Thus, there is an urgent and unmet need for a preclinical mouse model mimicking the patients, in which both the immunity and the tumor are of human origin and genetically identical. In this review, we first briefly overview the development and evolution of HIS mouse protocols, then discuss progress to date in creating HIS mice with autologous or genetically identical human tumors.

## Brief Review of HIS Mouse Construction

There has been a long-standing effort to create and optimize HIS mouse models. While different HIS mouse construction protocols have been reported, all involve transplantation of human hematopoietic and/or lymphoid cells (e.g., peripheral blood lymphocytes (PBLs), bone marrow cells, cord blood cells, or fetal liver hematopoietic cells) into immunodeficient mice. Current HIS mouse models are in general derived from three HIS mouse models reported in the late 1980s. Mosier and colleagues reported in 1988 that injection of human PBLs into C.B-17 severe combined immunodeficiency (SCID) mice resulted in durable reconstitution with human T cells, B cells and monocytes/macrophages, providing a useful model for the study of human immune function (known as hu-PBL-SCID mouse) (8). During the same period, McCune and colleagues reported another HIS mouse model (referred to as SCID-hu mouse by the authors), in which human immune reconstitution was achieved in C.B-17 SCID mice by transplantation of fetal thymus, liver and lymph node (9). Subsequently, using bg/nu/xid mice Kamel-Reid and Dick found that transplantation of human hematopoietic stem/progenitor cells (HSCs/HPCs) into immunodeficient mice could achieve human HSC/HPC engraftment and differentiation, offering an additional HIS mouse model (referred to as chimeric human/immune-deficient (HID) mice by the authors) (10). However, this HID model or similarly created HIS mouse models (i.e., constructed by human HSCs/HPCs of different sources) were not very useful for the study of human immunity due to poor T cell function until better immunodeficient mouse strains became available (see discussion below), and was further improved by using newborn immunodeficient mice as the recipients (11, 12). Despite these improvements, human T cells developing in the xenogeneic mouse thymus were increasingly reported to be functionally abnormal, likely caused by poor HLA-restricted antigen recognition (13–15).

The hu-PBL-SCID model is simple, but it needs to be cautious when using this model because of the potential of infused human T cells to induce xenogeneic graft-versus-host disease (GVHD) that may confound assessment of human immunity and result in human effector T cell anergy (16). Although there are apparent differences between xenogeneic and allogeneic GVHD, the hu-PBL-SCID model was found useful in the study of human GVHD pathogenesis (17). Xenogeneic GVHD was effectively prevented in the human fetal thymus/liver (Thy/Liv)-grafted SCID-hu model, in which the majority of human T cells developed *de novo* in the murine host and therefore, those reactive to mouse antigens were purged during the negative selection process (9). However, we found that, due to the lack of sufficient repopulation with human dendritic cells (DCs) and B cells, the SCID-hu mice were inefficient in mounting antigen-specific immune responses *in vivo* (18). To solve this problem, we developed a new protocol in which HIS mice were made by combined transplantation of human fetal thymus (under renal capsule) and CD34^+^ HSCs/HPCs (i.v.) (18, 19). The resultant HIS mice showed reconstitution with human T cells, B cells and DCs, and acquired the ability to mediate robust antigen-specific immune responses *in vivo* and reject pig xenografts (19–22). These human Thy/HSC-grafted HIS mice were also found able to mediate anti-viral responses and were termed BLT mice in some other studies (23, 24). A disadvantage of the Thy/HSC-based HIS mouse model is the need to use fetal tissues. Therefore, increasing efforts are currently undertaken to optimize the potential of animal thymi to support human thymopoiesis. However, until such animal becomes available, the Thy/HSC HIS mouse model will likely still be instrumental.

Regardless of which protocol is used to construct HIS mice, magnificent improvements in engraftment and function of human hematopoietic and lymphoid cells were made by using more sophisticated immunodeficient mouse strains, such as nonobese diabetic/LtSz-*scid*/*scid* (NOD/SCID) (25) and NOD-*scid IL2R*γ*^null^* (NSG) (26) mice. C.B-17 SCID mice, which were most commonly used in HIS mouse construction before the availability of NOD/SCID mice, have high complement activity that mediates antibody-independent rejection of xenogeneic cells (27). CD47 is ligand of SIRPα, an inhibitory receptor on macrophages and DCs, and its engagement with SIRPα inhibits phagocytosis and endocytosis (28, 29). In a xenogeneic transplant setting, the inability of donor CD47 to interact with the recipient SIRPα is an important mechanism triggering donor cell rejection by macrophages (30, 31). NOD/SCID mice lack hemolytic complement, and express signal regulatory protein (SIRP)α capable of cross-reacting with human CD47 (32). In addition, substantial effort was made to improve the engraftment, differentiation, survival, and function of human hematopoietic and lymphoid cells in immunodeficient mice by introducing human cytokines. The MISTRG mouse is a good example, in which human M-CSF, GM-CSF/IL-3, and TPO genes are knocked into their respective mouse loci (33). HIS mice made with MISTRG mice showed markedly improved development and function of human innate immune cells than those made with NSG mice (33). However, it is worth mention that HIS mice carrying transgenes of human immunostimulatory cytokines under a constitutive promoter, e.g., the SGM3-NSG mice expressing human SCF, GM-CSF, and IL-3, are prone to develop fatal disease characterized by activation and widespread tissue infiltration of human T cells and macrophages, and show significant elevation in human proinflammatory cytokines including IL-6, IL-18, IFN-γ, and TNF-α (34, 35). The vigorous proinflammatory responses would likely confound the evaluation of interested immune functions in these HIS mice, such as cytokine storm or cytokine release syndrome induced by immunotherapies (36).

## Construction of HIS Mice With Autologous Tumor

There has been an emerging effort in developing human tumor-bearing HIS mouse models. Both human tumor cell derived xenograft (CDX; immunodeficient mice grafted with human cancer cell line cells) and patient-derived xenograft (PDX; immunodeficient mice grafted with patient cancer cells) models were widely and successfully used in understanding oncogenesis and testing anti-cancer drugs (37), these models, however, were not useful in the study of cancer immunology or immunotherapy due to the lack of human immune system. A conceivable approach to solve this problem is to construct human tumor-bearing HIS mouse models, in which both the tumor cells and the immune system are of human origin, which permit assessment of tumor-associated immune responses and immunotherapies. However, a notable limitation of such human tumor-bearing HIS mouse models is that the tumor cells are allogeneic to human immune system, so anti-tumor immune responses are expected to be largely driven by allogenicity rather than tumorigenicity. Although co-transplantation of tumor cells with immune cells (e.g., PBMCs) from the same patient may resolve this issue, this model will also suffer the same problem as the hu-PBL-SCID model discussed above (i.e., xenogeneic GVHD and human effector T cell anergy).

### HIS Mice With Autologous Human Leukemia

**Figure 1:**
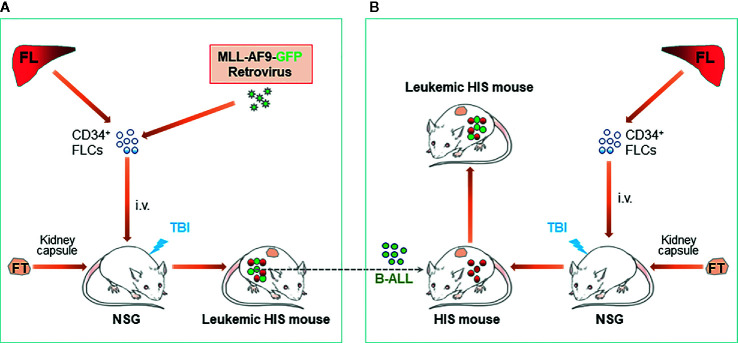
Construction of leukemic HIS mice with human immune system and leukemia derived from genetically identical HSCs/HPCs. **(A)** Schematic showing preparation of HIS mice with spontaneous development of B-ALL autologous to the human immune system. **(B)**. Schematic of leukemic HIS mouse construction by injection of autologous B-ALL [made as described in **(A)**] into preestablished HIS mice. FL, fetal liver; FLC, fetal liver cell; FT, fetal thymus; TBI, total body irradiation.

An ideal approach to resolving allogenicity would be to construct HIS mice with autologous tumor. Recently, we developed a HIS mouse model with spontaneous autologous leukemia and validated its usefulness in exploring anti-human leukemia immunotherapies. In this model, we transplanted sublethally-irradiated NSG mice with human fetal thymus and fetal liver CD34^+^ cells that were virally transduced with a mixed-lineage leukemia (MLL) fusion gene MLL-AF9 (**Figure 1A**) (38). It has been shown that MLL-AF9 expression drives the development of acute leukemia that resembles several clinical hallmarks of MLL leukemias (39). The NSG mice grafted with Thy/MLL-AF9-HSC appeared normal and showed a gradual increase in the levels of human PBMCs, including T cells, B cells and myeloid cells for about 3 to 4 months, then became progressively ill with a sharp increase in MLL-AF9-expressing HSC-derived CD19^+^ cells in blood. Autopsy revealed splenomegaly, enlarged lymph nodes, and hepatomegaly in all moribund mice. Histology demonstrated massive leukemic cell infiltration in bone marrow, spleen, lung, liver, and kidney. The MLL-AF9-expressing HSC-derived leukemic cells exhibited a high nucleus/cytoplasm ratio with a B-ALL phenotype, i.e., CD19^+^CD10^+^CD20^-^sIgM^low/-^sIgD^low/-^CD44^hi^MHC-I^+^MHC-II^hi^ and negative for other lineage markers, i.e., CD33^-^CD15^low/-^CD14^-^CD11b^-^CD3^-^CD4^-^CD8^-^CD56^-^. The study demonstrated that the Thy/MLL-AF9-HSC HIS mice not only develop human lymphohematopoietic cells, but also autologous B-ALL, offering a model to study human leukemia immunopathology and anti-leukemia immunotherapy in an autologous setting.

B-ALL cells developed in MLL-AF9-HSC-grafted HIS mice are transplantable in immunodeficient mice and in HIS mice with an established autologous human immune system (i.e., HIS mice made with human Thy/HSC from the same fetus from which CD34^+^ cells were used to develop the B-ALL) (38). However, rejection was seen when the B-ALL cells were transplanted in HIS mice with an allogeneic immune system. Adoptive transfer of cryopreserved B-ALL cells in pre-established HIS mice with an autologous immune system would present a much simpler model than spontaneous leukemia model described above (**Figure 1B**).

### HIS Mice With Autologous Human Solid Tumor

This model may also possibly be applied to set up HIS mice bearing autologous solid tumor. Although there has been no report of successful construction of autologous human solid tumor-bearing HIS mice, the feasibility of developing such HIS mouse models is supported by progress in understanding oncogenic changes causing tumorigenic transformation of normal cells. Previous studies were successful in inducing melanocytic transformation by engineering normal melanocytes to express a combination of specific mutations found in human melanoma, and the engineered human melanocytes could develop into melanocytic tumor in immunodeficient mice (40). Other studies identified combinations of oncogenic mutants that may drive tumorigenic transformation of human lung epithelial cells (41, 42). These studies suggested the possibility of establishing tumorigenic cells from normal fetal tissue cells. Successful generation of tumorigenic cells from fetal tissue cells would make it possible to construct HIS mice bearing autologous solid tumors by injecting the tumor cells into autologous HIS mice (i.e., HIS mice made with human Thy/HSC from the fetus from which parenchymal tissue cells are engineered for tumorigenesis). The feasibility of this approach is supported by a previous study, in which HIS mice were successfully used to assess the immunogenicity of autologous human induced pluripotent stem (iPS) cells (i.e., iPS cells reprogramed from fetal liver fibroblasts of the same fetus used for constructing HIS mice) (43).

### Personalized HIS Mice With Patient-Specific Immunity and Tumor

**Figure 2:**
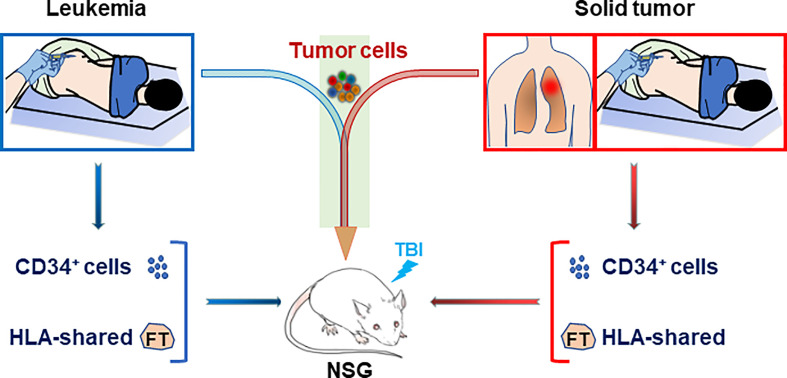
Construction of personalized HIS mice with patient-specific immunity and cancer. Personalized HIS mice are constructed by transplantation of CD34^+^ bone marrow cells (i.v.) from a patient bearing leukemia (Left) or solid tumor (Right) along with HLA allele-matched fetal thymic tissue (under renal capsule). The HIS mice will be followed for human immune reconstitution by flow cytometric analysis of blood cells and inoculated with the leukemic or solid tumor cells from the same patient when human immune reconstitution is confirmed. FT, fetal thymus; TBI, total body irradiation.

HIS mouse models were also used to study human immune function in a personalized manner, in which immunodeficient mice were grafted with patient-derived CD34^+^ bone marrow cells along with partially HLA allele-matched fetal thymic tissue (44). In this model, although human T cells were more “naïve” than those of the adult CD34^+^ cell donors, the immune recognition mimicked that of the adult donor, offering a model for individualized analysis of human immune function (44, 45). Furthermore, while the fetal thymus used was partially HLA-matched to the patient, human T cells developing in personalized HIS mice showed specific “self” tolerance (i.e., tolerance to the CD34^+^ cell donor patient). Combining this personalized HIS mouse with PDX model would provide a means of constructing patient-specific tumor-bearing HIS mice, in which both the tumor (leukemia or solid tumor) and immune system are derived from the same patient (**Figure 2**). Such a patient-specific tumor-bearing HIS mouse model should be highly valuable in personalized therapies.

## Modeling Anti-Leukemia Immunotherapy in HIS Mice With Autologous B-All

### Anti-Leukemic Responses Induced by Recipient Leukocyte Infusion

Following allogeneic hematopoietic cell transplantation (allo-HCT), donor T cells mediate beneficial graft-vs.-tumor (GVT) effects. However, allogeneic donor T cells also attack recipient normal tissues, resulting in GVHD. It has been reported that, in patients receiving nonmyeloablative allo-HCT, some of the patients who rejected donor grafts unexpectedly showed sustained remissions, suggesting an anti-donor alloresponse-associated antitumor activity (46). In support of this possibility, studies in mice found that administration of recipient leukocyte infusion (RLI) to mixed allogeneic chimeras results in rejection of donor hematopoietic chimerism and significant anti-host leukemia responses (47). RLI is apparently less effective than alloreactive donor T cells in killing recipient leukemia (48), but it does not induce GVHD, offering a potentially safe treatment for use in combination with other immunostimulatory therapies.

We have tested the potential of lymphopenia to enhance antitumor effects of RLI in leukemic HIS mice (**Figure 1**) (38). Lymphopenia is common in patients with leukemia who receive allo-HCT (49, 50), which is a factor that triggers GVHD (51, 52) but also promotes antitumor responses (53, 54). In this study, mixed chimeric (MC) HIS mice were established by transplantation of human Thy along with a mixture of ‘recipient’ (genetically identical to the Thy graft) and allogeneic ‘donor’ CD34^+^ HSCs, and lymphopenia was made by treatment with anti-huCD3-immunotoxin. Spleen cells from HIS mice made by transplantation of ‘recipient’ Thy/CD34^+^ cells were used as the RLI cells, which were autologous to the ‘recipient’ and allogeneic to the ‘donor’ origin of the MC HIS mice. Using this model, it was found that, in a human immune system, RLI was significantly more effective in inducing antitumor responses in lymphopenic than non-lymphopenic recipients, and that the antitumor response was associated with rejection of donor hematopoietic chimerism (38). The findings suggested that RLI offers a potentially safer clinical treatment option for leukemic patients who have profound lymphopenia.

### CD19-Targeted CAR T Cell Therapy

Recently, we made use of these leukemic HIS mice to model adoptive immunotherapy using human T cells that were genetically engineered to express anti-CD19 chimeric antigen receptors (CARs). CD19-targeted CAR T cell therapy has achieved promising results in patients with B-cell malignancies (55–57). However, despite the impressive response rates, many patients showed relapse or severe adverse reaction after anti-CD19 CAR T cell therapy (58, 59). Although memory CAR T cells were detected in patients (60), our understanding of these memory T cells, including their differentiation, function, self-renewal, and survival factors/signaling, remains limited. In addition, it remains largely unknown about the mechanisms responsible for the toxicities associated with anti-CD19 CAR T cell therapy, such as cytokine-release syndrome (CRS), which can be severe or even fatal (61). Thus, there is an urgent need to develop a preclinical model, which can be used to understand relapse and toxicity associated with human CAR T cell therapy, and to test the efficacy of new CAR T cells. Immunodeficient mice grafted with human B-ALL (PDX models) were found useful in testing CD19-targeted human CAR T cell therapy (62), but these models are either lacking host immunity or involving allo- and/or xeno-immune responses. Because the leukemic HIS mouse model described above has a functional human immune system and genetically-matched (autologous) primary B-ALL, this model was used to model CD19-targeted CAR T cell therapy (63). Another unique feature of this model is that the anti-CD19 CAR-expressing human T cells are also genetically-identical (autologous) to the human components (both normal and malignant human cells) and tolerant to mouse antigens of the HIS mice, and therefore do not mediate alloresponses against human or xenoresponses against mouse antigens. In leukemic HIS mice receiving CAR T cell therapy, the kinetics and levels of anti-CD19 CAR T cells in the peripheral blood were similar to those in patients (60). In agreement with clinical studies (55, 56), the frequency of CAR T cells in blood showed an inverse correlation with B-ALL burden but a positive correlation with survival times in CAR T cell-treated leukemic HIS mice. Moreover, this model was also found useful in characterizing cytokine profiles and regulatory T (Treg) cell generation and function following CAR T cell therapy. These observations provide a proof-of-principle that this leukemic HIS mouse model has the potential to be used to evaluate human CAR T cell therapy and help design new CARs with enhanced antitumor activity.

## Concluding Remarks

Apparently, the HIS mouse is not identical to a human host. However, there is strong experimental evidence for the value and feasibility of using HIS mice to study human immunity, including antigen-specific T cell and antibody responses (18–23, 64, 65). Thus, HIS mice with autologous tumors, either leukemia or solid tumor, would provide a highly valuable preclinical model for *in vivo* studies of human cancer immunology and immunotherapy.

## Author Contributions

All authors contributed to the article and approved the submitted version.

## Conflict of Interest

The authors declare that the research was conducted in the absence of any commercial or financial relationships that could be construed as a potential conflict of interest.
